# Long‐term outcome of ablation for anal HSIL: Achieving HSIL‐free status is key—A UK‐based retrospective cohort study

**DOI:** 10.1111/codi.70596

**Published:** 2026-09-15

**Authors:** T. Cuming, K. Rozemeijer, E. Farrow, C. Cappello, B. Wait, M. Junejo, J. Bowring, A. N. Rosenthal, O. Richel, M. Nathan, H. J. C. de Vries, J. M. Prins

**Affiliations:** ^1^ Homerton University Hospital London UK; ^2^ Imperial College London UK; ^3^ Department of Dermatology Amsterdam UMC Location University of Amsterdam Amsterdam The Netherlands; ^4^ Department of Pathology Amsterdam UMC, Location Vrije Universiteit Amsterdam Amsterdam The Netherlands; ^5^ Imaging and Biomarkers Cancer Center Amsterdam Amsterdam The Netherlands; ^6^ Sloane Kettering New York USA; ^7^ University College London Hospitals London UK; ^8^ Department of Infectious Diseases Radboud University Medical Center Nijmegen The Netherlands; ^9^ Infectious Diseases Amsterdam Institute for Infection and Immunity Amsterdam The Netherlands; ^10^ Department of Infectious Diseases, Center for Sexual Health Public Health Service Amsterdam Netherlands; ^11^ Department of Internal Medicine Amsterdam UMC Location University of Amsterdam Amsterdam Netherlands

**Keywords:** ablation techniques, anal cancer, anal neoplasms, cohort studies, follow‐up studies, high‐grade squamous intraepithelial lesions, HIV infections, laser therapy, precancerous conditions, sexual and gender minorities, squamous cell carcinoma

## Abstract

**Introduction:**

Treating anal high‐grade squamous intraepithelial lesions (HSIL) reduces squamous cell carcinoma (SCC) rates in high‐risk groups such as men having sex with men (MSM), especially those living with HIV (LWH). Follow‐up data after HSIL treatment is lacking.

**Method:**

A retrospective analysis was conducted of consecutive male patients with biopsy‐proven HSIL undergoing high resolution anoscopy (HRA)‐based ablation and subsequent surveillance at a UK specialist centre until either latest HRA or cancer diagnosis. The primary objective was the proportion becoming HSIL‐free after treatment.

Statistical analysis was carried out using time‐to‐event analysis with Cox proportional hazards and endpoints of anal cancer and HSIL recurrence.

**Results:**

A total of 136 men, 88% MSM, 78% LWH, underwent surveillance for a median of 5.5 (interquartile range (IQR) 4.0–6.8) years after laser ablation of 188 HSILs of the perianus (26%) or anal canal (74%).

After median 1 (IQR 1–2) ablations, 122/136 (90%) patients became HSIL‐free (definition: ≥ one HRA with cytology, free of HSIL), median 7 (IQR 5–14) months from index treatment. Fourteen of 136 (10%) patients never became HSIL‐free, of whom 5/14 (36%) developed SCC compared to 1/122 (0.8%) who were HSIL‐free at least once (*p* < 0.01). All six patients developing SCC were MSMLWH.

Never being HSIL‐free was associated with larger index lesions (*p* < 0.001) and a CD4 nadir of <200 (*p* < 0.001).

At study end, 98/130 without cancer had been HSIL recurrence free (75%) for median 31 (IQR 8–58) months, after total median two ablations (IQR 2–4).

**Discussion:**

HRA‐based treatment of anal HSIL with ablation allows the majority to become HSIL‐free. Closer surveillance is needed for those with persisting lesions.

**What does this paper add to the literature?**

This paper provides rare long‐term 5‐year follow‐up data after anal HSIL treatment, demonstrating higher progression to cancer in those whose disease is never cleared, but that the majority achieve cancer‐ and precancer‐free remission with 2–3 ablative treatments adding significantly to the knowledge‐base. UK papers on this topic are few.

## INTRODUCTION

Anal and perianal squamous cell carcinoma (SCC) incidence rates are rising worldwide [1] including key populations such as men who have sex with men (MSM) and those living with HIV (LWH) [2]. Anal high‐grade squamous intraepithelial lesions (HSIL) result from infection with oncogenic strains of human papillomavirus (HPV) [3, 4, 5]. HSIL and SCC are more prevalent in immunocompromised patients (e.g. those LWH), since they are more vulnerable to high‐risk HPV‐related oncogenesis [6]. Recent research has highlighted high resolution anoscopy (HRA)‐guided ablation of HSIL for SCC prevention, although much about treatment, follow‐up and recurrence remains to be determined [7, 8].

HRA is the standard for identifying anal HSIL and combines magnification of 30–50 times of the perianus (PA) and anal canal (AC) with chemical enhancement of squamous epithelium (5% acetic acid, Lugol's iodine) [9]. HSIL can be treated with ablation [10]. Electrocautery is widely used [7, 10, 11, 12, 13], although laser ablation [14, 15], established for vulval disease [16], infra‐red coagulation [17] and excision [18] are alternatives. While ablation is effective, studies have shown recurrence rates of 50%–77% [10, 11, 13, 17].

There is a paucity of evidence providing long‐term outcomes analysing recurrence rates after HSIL ablation and none from the United Kingdom. Data are observational and more than a decade old [10, 12, 14, 17]. The knowledge gap on the long‐term effectiveness of treatment is a barrier to the introduction of HRA and screening in the United Kingdom for high risk groups.

The current study was initiated to establish the outcome of male patients with HSIL treated by laser ablation at the largest referral centre in the United Kingdom.

### Objectives

The primary objective was to establish the rates of HSIL‐free (HF) status (treatment success) following treatment(s). Secondary objectives were to establish the factors affecting the achievement of HF status, rates of and factors affecting recurrence and cancer diagnoses, multizonal anogenital disease and time spent HF.

## METHOD

### Patients

This is a retrospective observational cohort study using prospectively collected data and electronic records. The Regional London Research and Ethics Committee, IRAS number 286346, approved the study.

HRA‐guided ablation was carried out for cases of consecutive males with histologically proven HSIL either referred from tertiary centres or diagnosed after the treatment of symptomatic warts from December 2014 to December 2019 and followed until January 2024 at Homerton Anogenital Neoplasia Service London. Cases with prior or prevalent anal cancer, no histological proof of HSIL or no post‐ablation HRA were excluded.

### Treatment and surveillance

Procedures were carried out under local anaesthetic (LA) in clinic, or general anaesthetic (GA) with laser ablation using either diode (Excel Lasers Ltd., Newmarket, UK) or CO_2_ laser (Lumenis Be Ltd., Yokeam, Israel). Post‐ablation HRA was carried out according to the unit's protocol (Figure S1) once the patient was HF, defined as cytology no more than LSIL (low grade squamous intraepithelial lesion) taken at the time of surveillance HRA which detected no lesions or no more than LSIL on biopsy.

Further disease ablated without confirmatory biopsy was considered recurrence. Cytology with moderate/severe dyskaryosis and negative HRA findings prompted recall as per guidelines [9].

High‐risk HPV testing (Roche Products Ltd., Basel, Switzerland) was introduced in 2019 on cytology samples.

### Data collection and definitions

The index lesion(s) was deemed to be the lesion(s) treated at the first ablation taking place after the start of the study period. Treatment of anal HSIL prior to the study period was noted but was not an exclusion.

Octants of AC and PA treatment were extracted as follows: a circle divided into equal eighths on tracing paper was overlaid on the contemporaneous AC/PA diagram in the paper notes and used to count the octants treated. Non‐contiguous lesions separated by at least one octant were considered separate lesions. Large index lesions totalled 5 octants or more [7]. Multiple lesions of varying sizes were considered an indicator of multifocality of disease, with octant number representing disease burden.

For large lesions, the index treatment date was the last date at which staged treatment (one treatment directly followed by another) was completed.

#### Definitions of treatment outcomes and recurrence

Treatment success was becoming HF, defined above. Recurrence was defined as a subsequent diagnosis of HSIL, either biopsy‐proven or treated without biopsy, after initial successful treatment. Recurrence and HF time were calculated from the date of the post‐ablation HF HRA until the earliest of the following events: HSIL recurrence, loss to follow‐up, death or latest HRA visit. Time to cancer diagnosis was calculated from the date of index treatment until anal cancer diagnosis.

### Multizonal disease

The term ‘multizonal’ was applied to patients with additional HPV‐related squamous intraepithelial lesions at genital sites and oropharyngeal SCC.

### Statistical analysis

Potential variables influencing treatment success were compared between patients who achieved HSIL clearance and those who did not (Table 1). Continuous variables were assessed for normality. Categorical variables were compared using the chi‐squared test when all expected cell counts were >5. Fisher's exact test was used for 2 × 2 tables with expected counts <5, and the Fisher–Freeman–Halton test was applied for tables with more than two categories when expected counts were <5. Cases with unknown values were excluded from statistical analyses.

Kaplan–Meier plots were used to visualise cumulative HSIL^+^ (ie HSIL+SCC) recurrence and cumulative SCC risk over time. For time‐to‐event analyses, Cox proportional hazards regression was performed to examine the associations between risk factors (age, MSM status, LWH status, CD4 nadir, immune compromised status, smoking history, presence of large lesions, number of lesions, areas affected, prior ablative or topical treatment and multizonal status) and HSIL^+^ recurrence risk. The log‐rank test was performed to compare the cumulative incidence of ASCC according to risk factors while accounting for varying follow‐up times.

Statistical analyses were performed with R studio software (version 2024.4.2+764; Posit, Boston MA); IBM SPSS Statistics software (version 25; IBM Corporation, Armonk, New York); and GraphPad Prism software (version 9.1.0; GraphPad Software, La Jolla, California). Reported *p* values are two‐sided, with 0.05 as the significance threshold.

To avoid bias, GA versus LA results and CO2 versus diode laser comparisons were avoided as categories were influenced by patient/clinician choice; adjuvant treatment with topical treatments likely represents harder‐to‐treat lesions.

Missingness was minimised by beginning data collection from the introduction of electronic data in 2014; by contacting patients and HIV consultants with permission for MSM and CD4 nadir data.

No artificial intelligence was used to generate any part of this manuscript.

## RESULTS

### Study population, lesions and treatment

#### Study population

**FIGURE 1:**
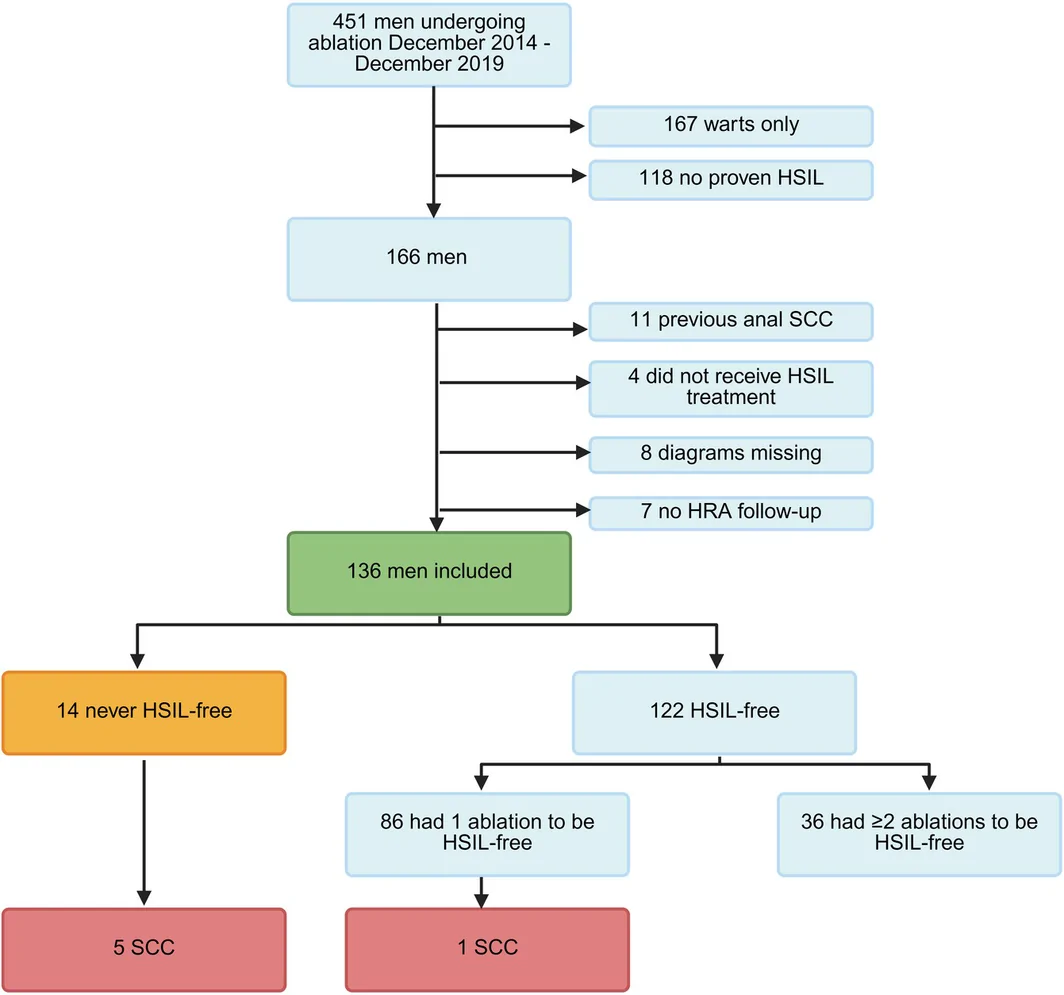
Patient flow diagram. From an initial 451 patients, 136 had histology‐proven HSIL with no known cancer at study initiation and adequate documentation. Created in https://BioRender.com. HSIL, high‐grade squamous intraepithelial lesion; SCC, squamous cell carcinoma.

Exclusions of 315/451 ablations during the study period (Figure 1) resulted in a final cohort of 136 patients undergoing ablation for biopsy‐confirmed HSIL of median age 47.5 (interquartile range (IQR): 39–55) years, with a median follow‐up of 5.5 (IQR: 4.0–6.8; minimum‐maximum/range: 0.3–8.9) years resulting in 711.7 person‐years (Figure 1). The majority of patients identified as MSM (120, 88.2%), and 106 (77.9%) were LWH at study entry for a median of 15 (IQR: 6–21) years. CD4 nadir was available for 88 patients, with a median of 193.5 (IQR: 42–372) cells/μL. Prior to study entry, 27 (19.9%) patients had prior ablative treatment and 25 (18.4%) topical treatment for HSIL (Table 1).

**TABLE 1:** Demographics of 136 men undergoing biopsy‐proven HSIL ablation (Homerton University Hospital 2015–2019).

Patients	*n* = 136
FU in years, median (IQR)	5.5 (4.0‐6.8)
Age in years, median (IQR)	47.5 (39.0‐55.0)
Sexual orientation, *n* (%)	
MSM	120 (88.2)
Heterosexual	7 (5.1)
Not known	9 (6.6)
Immunocompromised, *n* (%)^a^	116 (85.3)
LWH, *n* (%)	106 (77.9)
CD4 nadir in cells/μL, median (IQR)	193.5 (43–364.5); unknown for 18; n.a. for 30
Time LWH in years, median (IQR)	15 (6.5–21); unknown for 6; n.a. for 30
Treatment prior to study beginning, *n* (%)	
Ablation of HSIL	27 (19.9)
Topical treatment	25 (18.4)
Surface area of disease at index visit in number of octants, median (IQR)	3 (2–6)
Number of lesions, median (IQR)	1 (1–2)
Area affected, *n* (%)	
Perianal	20 (14.7)
Anal canal	91 (66.9)
Both	25 (18.4)
Multizonal HSIL+ disease, *n* (%)^b^	13 (9.6)
Smoking, *n* (%)	
Never	45 (33.1)
Ex‐smoker	32 (23.5)
Current	58 (42.6)
Unknown	1 (0.7)

*Note*: Continuous variables were assessed for normality. Normally distributed data are presented as mean ± standard deviation, whereas non‐normally distributed data are presented as median with IQR.

Abbreviations: HSIL, high‐grade squamous intraepithelial lesion; IQR, interquartile range; LWH, living with HIV; MSM, men who have sex with men; n.a., not applicable.

^a^ Comprising innate and pharmacological immune suppression states.

^b^ Multizonal = other HPV‐related malignant or premalignant disease of penoscrotal or oropharyngeal zones at study entry or developing during study.

#### Lesions

Ablation for biopsy‐proven HSIL was carried out on 188 index lesions in 136 men, of which 139/188 (74%) were AC and 49/188 (26%) PA; 81/188 were AIN3, 65 AIN2 p16 positive, and 31 AIN2 where no p16 was used, with 1 unknown.

Most patients (98, 72.1%) presented with a single lesion (IQR: 1–2). At the index visit, a median of 3 (IQR: 2–6) octants was affected. Large lesions were observed in 49/136 (36.0%), with a median of 6 (IQR: 6–8; range: 5–16) octants affected.

Five patients, all with large lesions (median 7 octants; range 5–16), underwent documented staged ablation becoming HF after a median of 14 (9–37) months. All were free of disease at study end for a median of 13 (0–44) months after a total of a median of 3 (2–7) ablations.

#### Treatment

More than half (105, 56%) of lesions were treated under LA with 44% under GA. Most ablations used diode laser (142, 75.5%; 132 AC vs. 10 PA), with 39 PA and 7 AC lesions treated by CO_2_ laser (46, 24.5%).

Complications occurred in 37 (27.2%) of patients, of whom 3 required intervention (Table S1) [19], including two stenoses which resolved (one needed dilatation), and 13 with moderate plus 3 with severe pain. Five deaths occurred, none of which were related to study interventions or anal cancer.

### Factors associated with acquiring HSIL‐free status

**FIGURE 2:**
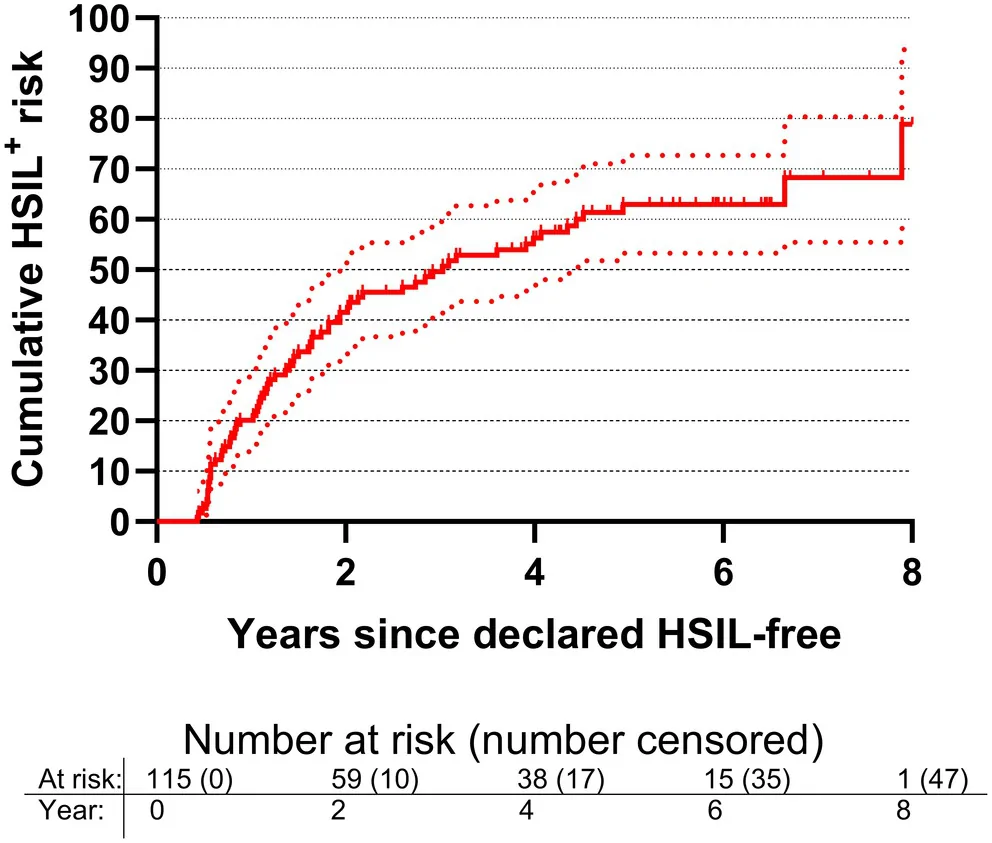
HSIL and SCC risk after receiving HSIL‐free status. Among 115 HSIL‐free patients with follow‐up, 67 developed HSIL, of whom one subsequently progressed to SCC thereafter. Dotted lines show the 95% CI, and vertical ticks indicate censored observations (last follow‐up without event). HSIL, anal high‐grade squamous intraepithelial lesions; SCC, squamous cell carcinoma.

**FIGURE 3:**
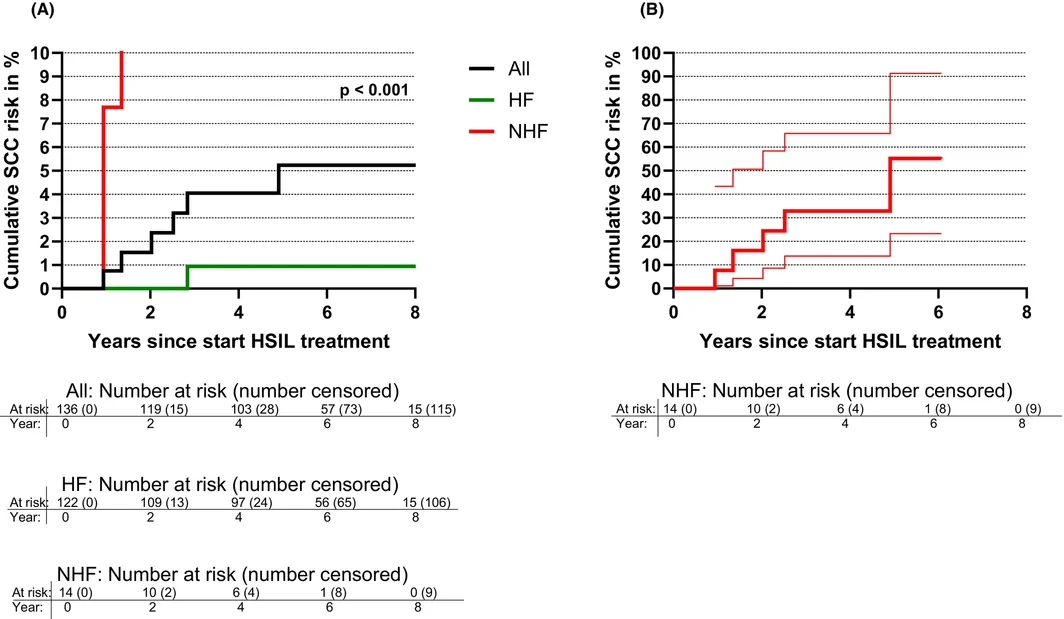
SCC risk after the start of ablation, stratified by achieving HSIL‐free status. Among 136 HSIL patients, six developed SCC after the start of ablative treatment. One case occurred in a patient who had achieved HSIL‐free status, whereas five cases occurred in patients who never achieved HSIL‐free status, in whom HSIL progressed to SCC despite treatment. (A) Cumulative SCC risk after the start of treatment is shown for all 136 HSIL patients, as well as stratified by patients who eventually achieved HSIL‐free status and those who did not. The log‐rank test was used to compare cumulative SCC risk between HSIL patient groups who eventually achieved HSIL‐free status and those who did not, while accounting for differences in follow‐up time. (B) Cumulative SCC risk after the start of treatment is shown for the 14 patients who did not achieve HSIL‐free status. The figure is scaled by a factor of 10 as compared with figure A. Light lines indicate the 95% confidence interval. HF, HSIL‐free; HSIL, anal high‐grade squamous intraepithelial lesions; NHF, never HSIL‐free; SCC, squamous cell carcinoma.

Of the 136 patients, 122 (89.7%) were HF after median 1 (IQR: 1–2; range: 1–6) ablations. The remaining 14 patients, who never became HSIL‐free (NHF), underwent median 2 (IQR: 2–4; range: 1–8) ablations. Among the NHF group, five (35.7%) developed SCC (Figures 2 and 3).

Factors associated with being NHF included large lesions (78.6% of NHF vs. 31.3% HF groups, *p* < 0.001) and low CD4 nadir (<200 cells/μL), present in 11/14 of those NHF (2 unknown nadir; 1 HIV negative) compared to 55.7% of those HF (68/122; 16 unknown) (*p* < 0.001). Prior treatment was non‐significantly protective (*p* = 0.08), with only 2 of 52 prior‐treated being NHF. No significant differences were observed between the NHF and HF groups with respect to other risk factors (Table 2).

**TABLE 2:** Comparison of characteristics between patients becoming HSIL‐free and patients never becoming HSIL‐free.

Variable	HSIL‐free (*n* = 122)	Never HSIL‐free (*n* = 14)	*p*‐Value
Age, *n* (%)			0.10
<45	49 (40.2)	3 (21.4)	
45–59	56 (45.9)	6 (42.9)	
≥60	17 (13.9)	5 (35.7)	
MSM, *n* (%)			
No	7 (5.7)	0 (0.0)	1.00
Yes	108 (88.5)	12 (85.7)	
Unknown	7 (5.7)	2 (14.3)	
Immunocompromised, *n* (%)^a^			
No	19 (15.6)	1 (7.1)	0.69
Yes	103 (84.4)	13 (92.9)	
LWH, *n* (%)			
No	29 (23.8)	1 (7.1)	0.30
Yes	93 (76.2)	13 (92.9)	
**Low nadir, *n* (%)**			
**No (≥200 or not LWH)**	**68 (55.7)**	**1 (7.1)**	**<0.001**
**Yes (<200)**	**38 (31.1)**	**11 (78.6)**	
**Unknown Nadir**	**16 (13.1)**	**2 (14.3)**	
Prior treatment (ablation or topical), *n* (%)			
No	72 (59.0)	12 (85.7)	
Yes	50 (41.0)	2 (14.3)	0.08
**Large lesions, *n* (%)**			**<0.001**
**No**	**84 (68.9)**	**3 (21.4)**	
**Yes**	**38 (31.1)**	**11 (78.6)**	
≥2 lesions, *n* (%)			0.21
No	90 (73.8)	8 (57.1)	
Yes	32 (26.2)	6 (42.9)	
Area affected			0.48
Perianal	19 (15.6)	1 (7.1)	
Intra‐anal	82 (67.2)	9 (64.3)	
Both	21 (17.2)	4 (28.6)	
Multizonal, *n* (%)			0.36
No	109 (89.3)	14 (100.0)	
Yes	13 (10.7)	0 (0.0)	
Smoking, *n* (%)			0.21
Never	41 (33.6)	4 (28.6)	
Ex‐smoker	26 (21.3)	6 (42.9)	
Current	54 (44.3)	4 (28.6)	
Unknown	1 (0.8)	0 (0.0)	

*Note*: Chi‐square tests were applied when all expected cell counts were greater than five. Fisher's exact test was used for 2 × 2 tables with expected counts less than 5, and the Fisher–Freeman–Halton test was applied for tables with more than two categories when expected counts were less than five. Cases with unknown values were excluded from statistical analyses. Variables were highlighted in bold if they differed significantly between patients who became HSIL‐free and those who never became HSIL‐free. Due to the limited number of events (i.e. patients who never became HSIL‐free), logistic regression could not be reliably performed for any of the variables.

Abbreviations: HSIL, anal high‐grade squamous intraepithelial lesions; LWH, living with HIV; MSM, men having sex with men.

^a^ Comprising innate and pharmacological immune suppression states.

Larger lesion size, low CD4 nadir, older age, multiple lesions and having PA with AC (PA + AC) disease were associated with an increased risk of either requiring >1 ablation to become HF or being NHF (Data S1).

### HSIL+SCC (HSIL ^+^) recurrence risk

Among 122 who became HF, 115 attended follow‐up (94.3%). Of these, 67 developed HSIL of whom one subsequently progressed to SCC. The cumulative risk of developing HSIL^+^ (i.e. HSIL or SCC) was 20.2% (95% CI: 12.8%–27.5%) within 1 year of HF, 41.5% (95% CI: 32.3%–50.8%) within 2 years, 63.0% (95% CI: 53.2%–72.8%) within five and 78.9% (95% CI: 59.9%–97.8%) within 8 years of becoming HF (Figure 2).

Factors significantly associated with HSIL^+^ recurrence included the presence of more than one lesion (hazard ratio (HR) 2.2; 95% CI: 1.3–3.7), PA + AC disease (HR of 2.6; 95% CI: 1.4–4.5) and being a current smoker (HR of 1.8; 95% CI 1.0–3.1). Needing multiple ablations to become HF seemed to be associated with an increased HSIL^+^ recurrence risk (HR of 1.6; 95% CI 1.0–2.7), albeit non‐significantly (Table 3). The effect of all four variables on the HSIL^+^ recurrence risk increased over time.

**TABLE 3:** Cox regression analysis: Variables associated with HSIL^+^ recurrence risk.

Variable	Hazard ratio (95% CI)	*p*‐Value
Age		0.91
<45	Reference	
45–59	1.03 (0.61–1.72)	
≥60	0.87 (0.39–1.92)	
Immunocompromised^a^		0.64
No	Reference	
Yes	1.17 (0.61–2.25)	
LWH		0.79
No	Reference	
Yes	1.08 (0.62–1.88)	
Low nadir		0.75
No (≥200 or not LWH)	Reference	
Yes (<200)	1.09 (0.65–1.83)	
Prior treatment (ablation or topical)		0.81
No	Reference	
Yes	0.94 (0.58–1.53)	
Large lesions		0.77
No	Reference	
Yes	1.08 (0.65–1.80)	
**≥2 lesions**		
**No**	**Reference**	**0.002**
**Yes**	**2.22 (1.33–3.72)**	
Area affected		
Intra‐anal	Reference	**0.001**
Perianal	0.76 (0.36–1.58)	
**Both**	**2.55 (1.44–4.50)**	
Multizonal		0.71
No	Reference	
Yes	1.15 (0.55–2.42)	
Smoking		
Never	Reference	0.07
Ex‐smoker	1.03 (0.48–2.20)	
Current	1.77 (1.03–3.06)	
Multiple ablations to become HSIL‐free		
No	Reference	0.06
Yes	1.62 (0.97‐2.72)	

*Note*: Variables were highlighted in bold if they differed significantly between patients who developed HSIL^+^ recurrence and those who did not.

Abbreviations: 95% CI, 95% confidence interval; HR, hazard ratio; HSIL^+^, anal high‐grade squamous intraepithelial lesions or SCC; LWH, living with HIV; MSM, men having sex with men; SCC, squamous cell carcinoma.

^a^ Comprising innate and pharmacological immune suppression states.

### Cancer risk

Six cancers occurred at mean 2.4 years (standard deviation: 1.4 years) from index ablation, of which five occurred in the 14 NHF patients (35.7%), compared to 1 SCC of the 122 HF located at 6 cm in the rectum within a rectal squamous island. Overall risk for progression to SCC 2 years after index treatment was 1.5% (95% CI: 0.0%–3.6%), 0.0% in the HF group and 16.1% (95% CI: 0.0%–36.6%) in the NHF. Equivalent figures at 5 years were 4.1% overall (95% CI: 0.6–7.5), 0.9% (95% CI: 0.0%–2.8%) in the HF compared to 55.2% (95% CI: 15.3%–95.2%) in the NHF (Figure 3).

Using the log‐rank test to account for differences in follow‐up time, the presence of multiple lesions, PA + AC disease and a low CD4 nadir were significantly associated with the development of SCC. Multiple lesions were present in 66.7% of patients who developed SCC versus 26.2% among those who did not (*p* = 0.03). Similarly, PA + AC disease was observed in 66.7% of patients who developed SCC versus 16.2% of those who did not (*p* = 0.01). Five of six (1 unknown) of those who developed SCC had a CD4 nadir <200 cells/μL compared with 33.8% (44/130; 17 unknown) of those without SCC (*p* = 0.01). Large lesions, MSM status, HIV‐infection, prior treatment status and smoking were not significantly associated with SCC development (Table 4).

**TABLE 4:** Comparison of characteristics between patients developing SCC and patients not developing SCC.

Variable	No SCC (*n* = 130)	SCC (*n* = 6)	*p*‐Value
Age, *n* (%)			0.15
<45	52 (40.0)	0 (0.0)	
45–59	58 (44.6)	4 (66.7)	
≥60	20 (15.4)	2 (33.3)	
MSM, *n* (%)			
No	7 (5.4)	0 (0.0)	0.55
Yes	114 (87.7)	6 (100.0)	
Unknown	9 (6.9)	0 (0.0)	
Immunocompromised, *n* (%)^a^			
No	20 (15.4)	0 (0.0)	0.28
Yes	110 (84.6)	6 (100.0)	
LWH, *n* (%)			
No	30 (23.1)	0 (0.0)	0.16
Yes	100 (76.9)	6 (100.0)	
**Low nadir, *n* (%)**			
**No (≥200 or not LWH)**	**69 (53.1)**	**0 (0.0)**	**0.01**
**Yes (<200)**	**44 (33.8)**	**5 (83.3)**	
**Unknown Nadir**	**17 (13.1)**	**1 (16.7)**	
Prior treatment (ablation or topical), *n* (%)			
No	79 (60.8)	5 (83.3)	
Yes	51 (39.2)	1 (16.7)	0.29
Large lesions, *n* (%)			0.10
No	85 (65.4)	2 (33.3)	
Yes	45 (34.6)	4 (66.7)	
**≥2 lesions, *n* (%)**			**0.03**
**No**	**96 (73.8)**	**2 (33.3)**	
**Yes**	**34 (26.2)**	**4 (66.7)**	
**Area affected**			**0.01**
**Perianal**	**20 (15.4)**	**0 (0.0)**	
**Intra‐anal**	**89 (68.5)**	**2 (33.3)**	
**Both**	**21 (16.2)**	**4 (66.7)**	
Multizonal, *n* (%)			0.43
No	117 (90.0)	6 (100.0)	
Yes	13 (10.0)	0 (0.0)	
Smoking, *n* (%)			0.62
Never	44 (33.8)	1 (16.7)	
Ex‐smoker	30 (23.1)	2 (33.3)	
Current	55 (42.3)	3 (50.0)	
Unknown	1 (0.8)	0 (0.0)	

*Note*: The log‐rank test was used to compare cancer‐free survival between groups while accounting for differences in follow‐up time. Cases with unknown values were excluded from statistical analyses. Variables were highlighted in bold if they differed significantly between patients who developed SCC and those who did not develop SCC. Due to the limited number of ACC, regression analyses could not be reliably performed for any of the variables and statistical power of the log‐rank test was limited.

Abbreviations: LWH, living with HIV; MSM, men who have sex with men; SCC, squamous cell carcinoma.

^a^ Comprising innate and pharmacological immune suppression states.

### End of study status

Recurrence occurred after becoming HF, requiring additional treatment. Median total ablations during the study period were 2 (IQR: 1–3; range 1–18), supplemented with topical treatments in 44/136 (32.4%) and HRA‐guided excision in 21/136 (15.4%).

Of the 130 patients without cancer, 98/130 (75%) had no detectable HSIL for median 31 (IQR: 8–58) months prior to study end. This includes 17 cases whose final HRA confirmed no HSIL after a recent treatment for recurrent disease (recorded as 0 months clear of HSIL). Of the others, 81/130 (62%) had been clear of HSIL recurrence at study end for median 43 (IQR: 19–64) months, with 55/130 (45%) clear for >1.5 years and 22/130 (13%) clear for >5 years.

High‐risk HPV testing available at study‐end surveillance HRA in 101/130 without cancer was negative in 27 (27%) and detectable in 74 (57%), of which 20 were HPV 16 positive.

### Multizonal disease

We noted a multizonal disease history for 12 patients (12 one additional zone, 1 with 2 additional zones): HSIL at 7 penile, 1 rectal (in metaplastic squamous epithelium) and 2 natal cleft sites, in addition to 1 scrotal SCC and 2 oropharyngeal SCCs.

Multizonal disease occurred in 2/9 HIV‐negative immunosuppressed men (22.2%).

## DISCUSSION

This study over a median of 5.5 (IQR: 4.0–6.8; range: 0.3–8.9) year surveillance period demonstrates that HSIL ablative treatment in a male cohort referred to a specialist unit results in HF status for around 90% of patients (122/136). This referral‐based cohort differs from screened cohorts in the literature, potentially presenting more advanced or treatment‐resistant disease. For example, the ANCHOR study reported extensive (≥5 octant of anal or perianal region) disease in 13% of patients, compared to 36% (49/136) in this cohort [7]. Although 56% of the 88 patients with available CD4 nadir data had counts <200 cells/μL, similar to the ANCHOR study's 51% ≤200/μL, prior HSIL treatment before study entry occurred in 28% of our cohort versus 10% in ANCHOR.

Achieving HF status appears to be significant, with the cancer detection rate of 36% (5/14) in patients who never cleared HSIL, compared to one HPV‐associated rectal squamous cancer of 122 that became HF. HRA may potentially miss rare rectally located lesions due to a short straight anoscope, but most should be palpable at digital anorectal examination. The cancer rate for the ever HF group at 2 years was similar to that in the ANCHOR study at 25.8 months of 0.9% in the treatment arm (vs. 1.8% in the active monitoring arm) [7].

Three of the 14 (21%) never HSIL‐free cases failed to attend after an HRA visit confirmed recurrent HSIL, with outcome unknown. It is possible that in the five patients with persistent HSIL who developed cancer, early cancer had been missed at one of their HRA visits. Nonetheless, HRA‐based surveillance enabled diagnosis at Stage 1 (T1N0 or SISCCA) in four of five, compared to the UK average of 11% [20]. When persistent HSIL is recognised, HRA practitioners should inform patients and arrange vigilant follow‐up. It may not be possible to prevent all SCCs; however, it is recommended that practitioners discuss challenging cases with colleagues and continue to offer treatments.

While cancer risk and HSIL recurrence correlated with known anal cancer risk factors, correlation with higher lesion number and HSIL at ‘PA + AC’ sites may indicate multifocality, potentially another marker of increased risk.

Large lesions usually require multiple treatment visits [7], however, several patients underwent staged ablation and successfully cleared extensive HSIL lesions without developing SCC. Topical treatments were used adjuvantly for downstaging extensive disease and for recurrence, and as is common in the UK, 25 patients had topicals prior to referral.

This data is the longest follow‐up (66 months) of any post‐treatment observational dataset in the literature, and demonstrates that recurrence after HF status occurs steadily with only 21% recurrence‐free at 8 years.

### Comparison with other studies

Palefsky et al demonstrated strong lesion size influence on cancer risk in ANCHOR, with incidence of 1047/100,000 person‐years for large (>50% of any anal zone) lesions versus 185/100,000 for smaller lesions over 24.5 months of observation [7].

This study is an assessment of HSIL ablation in a real‐world setting. Other studies of ablation also show significant levels of recurrence after multiple treatments, although we here differentiate true recurrence as occurring after HF status (i.e. successful treatment) is established. Richel et al. found 71% recurrence at 76 months [10] and Gaisa et al. 60% at 12 months [11]. In addition, five patients within Goldstone's large observational dataset of 722 developed cancer despite treatment [14]. Even in the best hands, HSIL treatment will not prevent all cancers, but will reduce incidence and decrease stage [7, 14].

### Strengths and limitations

This study's strength lies in its long surveillance period (median 66 months) with comprehensive follow‐up in a real world setting: inclusion criteria required at least one follow‐up HRA, with only 20% not completing follow‐up to study end. This may be helpful in a field where understanding long‐term outcomes has proven difficult. The referral case‐finding and subsequent severity of disease presented may limit generalisability to cases of screen‐detected HSIL; however, this represents current UK anal HSIL presentation, where screening is not yet instituted.

The retrospective design creates limitations: missing CD4 nadir data (some unobtainable); because only six participants developed cancer during follow‐up, the statistical power of the log‐rank test was limited, and the results should therefore be interpreted with caution; including patients with prior treatment may have underestimated the number of ablations to become HF, resulting in inaccurate associations for some variables; lack of documented biopsy‐proven HSIL in the original larger cohort leading to patients lost to the study; potential overestimation of recurrence by assuming all further treatment without histology was recurrence; provider HRA expertise limiting generalisability; and HSIL over‐diagnosis prior to routine p16 immunohistochemistry. Counting HF time from first HF HRA instead of index treatment may have underestimated HF time, but was chosen for clarity.

Establishing HF status depends on HRA quality. An expert with 30 years' experience (MN) trained the other nine anoscopists involved; all attended HRA courses, underwent in‐house certification, and carry out 100 to 700 HRAs per year. However, this study preceded any national/international HRA certification process. Furthermore, it is a single‐centre study albeit with a sizeable team.

The 4–6 month intervals between assessments/treatments limit the survival curves shown.Recurrence/persistence could theoretically be detectable a few weeks later but only identified at the 4–6 months assessment.

### Meaning of the study

This study provides additional information clinicians can discuss with patients at HSIL diagnosis. For younger patients without immunosuppression or with smaller lesions, 1–2 ablations should achieve HF status lasting an average 16 months, and just over half will need 1–2 further treatments within 5 years.

Clinicians should identify those at higher risk of recurrence and cancer: older patients, smokers and the immunosuppressed (especially if low CD4 nadir), those with larger lesions or evidence of multifocality.

Biomarker future research may help predict the group (55/122, 45%) who maintained long‐term HF status (median 4.3 years) without recurrence [21] enabling earlier cessation of surveillance. Researchers should further study multizonal disease in men: 9% of this cohort but an additional 25 with PA + AC disease, as a potential marker of HPV immune susceptibility.

## CONCLUSION

Although there is a need for multiple HSIL ablations due to recognised high recurrence rates, treatment programmes should be geared towards achieving HF status. HSIL resistant to treatment should be an alert to either an underlying occult cancer or its imminent development. This study provides long‐term data that HSIL ablation can induce and maintain HSIL remission in most patients, even in severely affected cohorts.

## AUTHOR CONTRIBUTIONS

**K. Rozemeijer:** Methodology; visualization; formal analysis; data curation; software; writing – review and editing; validation. **H. J. C. de Vries:** Supervision; writing – review and editing; methodology. **M. Junejo:** Investigation; methodology. **O. Richel:** Writing – review and editing; supervision; methodology. **J. M. Prins:** Supervision; writing – review and editing; methodology. **J. Bowring:** Writing – review and editing; conceptualization. **T. Cuming:** Conceptualization; investigation; writing – original draft; methodology; data curation; writing – review and editing; formal analysis; validation; visualization; project administration. **E. Farrow:** Investigation; methodology; writing – original draft. **B. Wait:** Investigation; methodology. **M. Nathan:** Conceptualization; investigation; writing – review and editing; methodology; supervision. **C. Cappello:** Investigation; writing – review and editing; writing – original draft. **A. N. Rosenthal:** Writing – review and editing.

## FUNDING INFORMATION

No funding source was utilised for this study.

## CONFLICT OF INTEREST STATEMENT

M Nathan declares 2025 travel and honorarium by Protocom; no conflicts of interest have been declared by any other author.

## ETHICS APPROVAL STATEMENT

This study underwent ethics approval of the London Research and Ethics Committee with IRAS number 286346.

## PATIENT CONSENT STATEMENT

Retrospective data was used, with no specific patient consent obtained. This was approved by the ethics committee.

## PERMISSION TO REPRODUCE MATERIAL FROM OTHER SOURCES

No material was used from other sources except for the visual abstract which was constructed via a website.

## Supporting information


**Figure S1.** Available at: https://www.homerton.nhs.uk/hans‐professional‐resources.
**Table S1**. (S1) Complications, Mortality of all ablations December 14–December 2019, Homerton, established by telephone follow‐up at 1 week and routine clinical review.
**Table S2**. Comparison of characteristics between patients becoming HSIL‐free after one ablation versus patients never becoming HSIL‐free or patients needing multiple ablations to become HSIL‐free.

**Data S1.** STROBE Statement—Checklist of items that should be included in reports of *cohort studies*.

## Data Availability

The data that support the findings of this study are available from the corresponding author upon reasonable request.
