# Supplementary material for: Long‐term outcome of ablation for anal HSIL: Achieving HSIL‐free status is key—A UK‐based retrospective cohort study
Source: Colorectal Dis. 2026 Sep 15;28(9):e70596. doi: 10.1111/codi.70596 (PMC13578555; doi:10.1111/codi.70596)
Supplement: Supplementary file 1 [file CODI-28-0-s001.docx]

**Supplementary Figure 1**

Available at: https://www.homerton.nhs.uk/hans-professional-resources

**Supplementary Table 1 (S1) Complications, Mortality of all ablations Dec 14-Dec 2019, Homerton, established by telephone follow up at 1 week and routine clinical review.**

| **Complications n = 37** | **Numbers affected** | **Clavien-Dindo classification** |
| --- | --- | --- |
| Moderate (13) to severe pain (3)* | 16 | Grade 1 |
| Miscellaneous: perianal haematoma, fissures (4), UTI (2), mucus discharge for 3/12, sensation of scar, urinary retention overnight catheter | 10 | Grade 1 |
| Stenosis – one required self-dilation for 4 months; other resolved | 2 | Grade 1 |
| Faecal incontinence - mild/self-limiting no treatment required | 5 | Grade 1 |
| Bleeding – A&E tranexamic acid | 1 | Grade 2 |
| Chronic fissure – requiring GA Botox | 1 | Grade 3b |
| Fistula – one prior fistula reopened by treatment; | 1 | Grade 3b |
| Pulmonary Embolus** | 1 | Grade 4a |
| **Deaths** | **Numbers** | **Causes** |
|  | 5 | Lung cancer, Merkel cell tumour, suicide, cardiac event, accidental drug overdose. |

*Lasting more than 1 week or requiring narcotic analgesia: occurred prior to introduction of staged ablation; all but 1 were large ablations of >50% of anal canal in one sitting, modified after 2018 to staged treatments **Incident occurred 10 days after local anaesthetic procedure in clinic of 3 octant ablation.

**Supplementary Table S2. Comparison of characteristics between patients becoming HSIL-free after one ablation *versus* patients never becoming HSIL-free or patients needing multiple ablations to become HSIL-free.**

| **Variable** | **HSIL-free after one ablation (n=86)** | **Never HSIL-free or HSIL-free needing multiple ablations (n=50)** | **p-value** |
| --- | --- | --- | --- |
| **Age, n (%)**  **<45**  **45-59**  **≥60** | **37 (43.0%)**  **40 (46.5%)**  **9 (10.5%)** | **15 (30.0%)**  **22 (44.0%)**  **13 (26.0%)** | **0.05** |
| MSM, n (%)  No  Yes  Unknown | 3 (3.5%)  79 (91.9%)  4 (4.6%) | 4 (8.0%)  41 (82.0%)  5 (10.0%) | 0.24 |
| †Immunocompromised, n (%)  No  Yes | 14 (16.3%)  72 (83.7%) | 6 (12.0%)  44 (88.0%) | 0.62 |
| LWH, n (%)  No  Yes | 19 (22.1%)  67 (77.9%) | 11 (22.0%)  39 (78.0%) | 1.00 |
| **Low nadir, n (%)**  **No (≥200 or not LWH)**  **Yes (<200)**  **Unknown Nadir** | **51 (59.3%)**  **25 (29.1%)**  **10 (11.6%)** | **18 (36.0%)**  **24 (48.0%)**  **8 (16.0%)** | **0.01** |
| Prior treatment (ablation or topical), n (%)  No  Yes | 53 (61.6%)  33 (38.4%) | 31 (62.0%)  19 (38.0%) | 1.00 |
| **Large lesions, n (%)**  **No**  **Yes** | **62 (72.1%)**  **24 (27.9%)** | **25 (50.0%)**  **25 (50.0%)** | **0.02** |
| **≥2 lesions, n (%)**  **No**  **Yes** | **74 (86.0%)**  **12 (14.0%)** | **24 (48.0%)**  **26 (52.0%)** | **<0.001** |
| **Area affected**  **Peri-anal**  **Intra-anal**  **Both** | **13 (15.1)**  **65 (75.6)**  **8 (9.3)** | **7 (14.0%)**  **26 (52.0%)**  **17 (34.0%)** | **0.001** |
| Multizonal, n (%)  No  Yes | 79 (91.9%)  7 (8.1%) | 44 (88.0%)  6 (12.0%) | 0.55 |
| Smoking, n (%)  Never  Ex-smoker  Current  Unknown | 34 (39.5%)  16 (18.6%)  35 (40.7%)  1 (1.2%) | 11 (22.0%)  16 (32.0%)  23 (46.0%)  0 (0.0%) | 0.07 |

Chi-square tests were applied when all expected cell counts were greater than 5. Fisher’s exact test was used for 2×2 tables with expected counts less than 5, and the Fisher–Freeman–Halton test was applied for tables with more than two categories when expected counts were less than 5. Cases with unknown values were excluded from statistical analyses. Variables were highlighted in bold if they differed significantly between patients who became HSIL free and those who never became HSIL-free.

†Comprising innate and pharmacological immune suppression states.

Abbreviations: LWH= living with HIV; MSM= men who have sex with men;
